# From slacktivism to activism: Improving the commitment power of e-pledges for prosocial causes

**DOI:** 10.1371/journal.pone.0231314

**Published:** 2020-04-29

**Authors:** Eileen Y. Chou, Dennis Y. Hsu, Eileen Hernon

**Affiliations:** 1 Batten School of Leadership and Public Policy, University of Virginia, Charlottesville, Virginia, United States of America; 2 Faculty of Business and Economics, The University of Hong Kong, Hong Kong, Republic of China; 3 University of Virginia, Charlottesville, Virginia, United States of America; Middlesex University, UNITED KINGDOM

## Abstract

Prosocial organizations increasingly rely on e-pledges to promote their causes and secure commitment. Yet their effectiveness is controversial. Epitomized by UNICEF’s “Likes Don’t Save Lives” campaign, the threat of slacktivism has led some organizations to forsake social media as a potential platform for garnering commitment. We proposed and investigated a novel e-pledging method that may enable organizations to capitalize on the benefits of e-pledging without compromising on its mass outreach potential. In two pilot studies, we first explored whether and why conventional e-pledges may not be as effective as intended. Building on those insights, we conducted one field and two lab experiments to test our proposed e-pledge intervention. Importantly, the field study demonstrated the effectiveness of the intervention for commitment behavior across a 3-month period. The laboratory experiments provided a deeper and more refined mechanism understanding of the effect and ruled out effort, novelty, and social interaction mindset as alternative explanations for why the intervention may be effective. As technological innovations continue to redefine how people interact with the world, this research sheds light on a promising method for transforming a simple virtual acknowledgment into deeper commitment—and, ideally, to action.

## Introduction

In 2014, Indonesian political analyst Denny Januar Ali amassed more than 2.5 million retweets that pledged to support Indonesian presidential candidate Joko “Jokowi” Widodo and to replace discrimination with love [1]. In 2016, Facebook COO Sheryl Sandberg rallied the platform’s 1.6 billion users to redirect their 6-billion-times-a-day habit of clicking “Like” by supporting an online campaign committed to defeating ISIS recruiters [2]. The power of social media as an efficient and massive information dispenser testifies to its capacity to mobilize support on an unprecedented level. Proponents of using social media as a key campaign platform also tout its potential trickle-down effect: By raising awareness, people are more likely to engage in activities that may indirectly help the given cause. As a result, political and social groups increasingly dedicate resources to these new online efforts [3].

Despite the rapid growth of social media campaigns, the notion of slacktivism—defined as “feel-good online activism with little meaningful social or political impact” [4–5]—challenges the value of these efforts. Slacktivism highlights the ease with which people can “click it and forget it.” Based on this assumption, some even argue that engaging in online campaigns may lead people to believe that they have already contributed to the cause, without doing anything meaningful. Indeed, a recent poll revealed that only 3% of active social media users cited online campaigns as a key motivator in their donation decisions [6]. In a field study in collaboration with Heifer International, Lacetera, Macis, and Mele showed that an online campaign engaged almost 6.4 million online users (in the form of “likes” or “shares”), yet only 30 made an actual donation [7]. The concern that slacktivism could encroach on tangible support has led, for instance, to UNICEF Sweden’s “Likes Don’t Save Lives” campaign [8].

Rather than focusing on an impact evaluation of online campaigns, we contend that the more pressing and realistic issue is how we can improve the existing platform to secure greater commitment, thereby allowing organizations to capitalize on the platform’s power. With this goal in mind, we focused on e-pledges—one of the most common methods used by online campaigns—and shed light on the prevalent phenomenon of slacktivism. We aimed to answer two questions: (a) why are e-pledgers less motivated to follow through on their pledges, and (b) what type of intervention might increase e-pledges’ commitment power?

We conducted five studies to tackle these questions, with the results of each study informing the next. Pilot Study 1A demonstrated the presence and prevalence of slacktivism by directly comparing the effectiveness of conventional e-pledges with their traditional counterparts. Pilot Study 1B provided a layperson’s perspective as to why conventional e-pledges are ineffective. These insights then served as the foundation for a novel e-pledging method that seeks to strengthen pledgers’ commitment to prosocial causes. Based on the aggregated results, we reasoned that e-pledges that can better activate the pledger’s sense of public self-awareness and personal accountability [9] would be more effective for securing commitment behavior. Therefore, we built on the existing literature and tested our prediction—that instructing participants to pledge with both their own name and those of someone important to them—would be an effective e-pledge intervention. Study 1, a field experiment, tested the effect of the intervention against two common forms of conventional e-pledging methods: clicking on the “Like” icon (as often used on social media platforms) and typing their name. Results from Study 1 then prompted us, in Studies 2 and 3, to explore explanations why the intervention might work and rule out alternative explanations [10]. We present all measures in this paper and data analyses occurred after the predetermined data collection period (see online supplemental material for the actual experimental materials).

This research offers insights with potential practical and theoretical advancements. First, we empirically investigated a promising solution to a pervasive problem: the increasing, yet ineffective, reliance on e-pledges as a way to secure prosocial commitment. By revealing laypeople’s perspectives on why e-pledges might contribute to slacktivism, the aggregated trend in our data played a crucial role in developing an ecologically valid intervention. Second, we ruled out several closely related alternative explanations to the effectiveness of the intervention. By investigating both whether the intervention would work and why, our research lays a foundation for future research to generate flexible and improved e-pledge methods. In turn, this work augments the scope of its practical implications. Third, this paper integrates theoretical perspectives on social influence and objective-self-awareness, and presents a set of empirically driven research questions for future studies. Lastly, given the continuing surge of social media, a rapidly changing demographic, and e-pledges’ potential to reach a broad audience, our research aims to shed light on how to effectively transform virtual acknowledgement into deeper commitment—and, ideally, action.

### Understanding what e-pledging is and the source of its ineffectiveness

A pledge is a person’s solemn promise to commit to a cause that he or she deems worthy [11]. In essence, pledges serve as a means of social control. Yet unlike formal contracts or sanctions, failure to honor a pledge has minimal punitive consequences. Therefore, the commitment power of pledges often relies on (a) the normative expectation that people will be held accountable after making the pledge [12] and (b) self-investment and identification with the cause [4, 13, 14]. Together, these critical forces motivate individuals to fulfill their pledge.

People traditionally confirm their pledge by signing their name on a piece of paper or publicly announcing their commitment to the cause. An e-pledge, which we define as a virtual promise to honor a commitment, serves the same objective function as traditional pledges. The only substantive difference is the method by which people pledge: Instead of signing their name by hand on paper, would-be-pledgers indicate their commitment electronically, either on a social media platform (e.g., Facebook, Twitter) or through an online portal (e.g., Change.gov, Redcross.org).

However, as a plethora of slacktivism anecdotes suggest, e-pledges may not be as effective in their ability to secure commitment as their traditional counterparts. We posit that while e-pledges and traditional pledges serve the same objective functions, they diverge in the psychological weight they may evoke in the pledger. Indeed, past research posits two potential drivers of this ineffectiveness: (a) The online pledge in general is perceived to be less trustworthy or mobilizing (regardless of how it was signed) or (b) the method used to pledge (e.g., “Like” clicking, name initials typing, etc.) dilutes the commitment effect. We expand on these two drivers below.

On the one hand, it could be that people consider online pledges to be less persuasive or trustworthy. In line with this notion, prior research has found that people perceive electronically transmitted documents to be less trustworthy [15]. Therefore, it could be that people are willing to pledge their support, but question whether the campaign itself warrants further involvement. As a result, they stop short of actual action.

On the other hand, it could be that the pledging process itself is less effective for motivating people to take the desirable action. In recent e-signature research, compared with participants who signed by hand, e-signers were less likely to obey the terms of the contract they signed [16]. Similarly, consumers who typed their names (versus signing by hand) were less likely to make a purchase afterward [17]. These findings suggest that conventional methods of e-pledging may be the reason for its ineffectiveness, independent of the cause or campaign being promoted.

To address slacktivism within the e-pledge domain, we first need to demonstrate empirically that it is indeed an issue and then try to understand the underlying source and mechanism of the problem. To this end, we conducted two pilot studies that served two purposes. First, Pilot Study 1A and 1B s provided an empirical assessment of laypeople’s engagement in and perception of slacktivism—specifically, its presence, prevalence, and severity. Second, we presented participants in Pilot Study 1B with the two potential sources of e-pledge’s ineffectiveness grounded in prior research, and gauged which they deemed more consequential. Based on the results, we were able to derive the most suitable way to improve the overall effectiveness of e-pledging for securing commitment behaviors.

## Pilot Study 1A: Are conventional e-pledges effective?

Pilot Study 1A set out to demonstrate whether conventional e-pledges are indeed less effective than the traditional way of pledging in a prosocial domain that supports scientific advancement. To do so, we investigated whether three different forms of pledging could influence subsequent commitment behavior in variant degrees. We included two common forms of e-pledging—a checked box (as often seen on platforms such as Facebook and Twitter) and typed full name (as often seen on platforms such as Change.gov and Redcross.org)—and compared their impact to the traditional way of pledging with a handwritten signature. We then measured their subsequent commitment behavior to the clause.

### Methods

#### Participants and procedure

Ninety-three undergraduate students (mean age = 20.38, *SD* = 3.63; 51% female) participated in the study in exchange for a snack and the chance to win a $50 bonus. We obtained IRB approval from the University of Virginia to conduct this study, with written consent from the participants.

Participants completed a two-stage study on a laptop preloaded with the study programed in Qualtrics. In the first stage, participants were informed of a cover story that the study was interested in decisions made under time pressure. They then played three rounds of “Where’s Waldo?.” Each round presented participants with a large image and asked them to locate the figurine “Waldo.” Participants had up to 30 seconds per round to find Waldo. We included this first stage and a cover story to minimize potential demand effect of participants succumbing to how they think the experimenter would want them to behave.

#### Pledge-signing manipulation

Upon completing the Where’s Waldo task, participants then learned that they would have the option to sign a pledge to support evidence-based behavioral research at their institution. The pledge read as follows:

*Please read the following petition regarding behavioral scientific research*, *and sign if you agree*. *Otherwise*, *leave this blank and move to the next page*.

*To create a better tomorrow*, *we must start today and draft evidence-based policies*. *Investing time*, *focus*, *and money in understanding the social and psychological implications of public and private policies is crucial in their eventual effectiveness*.

*Join us at the Behavioral and Science Policy Association (behavioralpolicy*.*org) in helping to develop a rigorous*, *comprehensive*, *and evidence-based behavioral research*. *No matter what you do*, *let your actions be seen*.

Participants were then randomly assigned to sign the pledge in one of three ways. Participants were asked to either “*Take the pledge by click on the Like button below*” (Like condition), “*Take the pledge by typing your initials below*” (initials condition), or “*Take the pledge by signing your name with the cursor in the space below*” (traditional pledge condition). Everyone read the exact same pledge. The only difference was how they signed the pledge.

#### Commitment behavior

After the pledge, participants were told that the experimenters would like to gain insight into how to improve the participant-recruitment process at their institution. Their responses would allow the experimenters to enhance behavioral research. Participants were then given the opportunity to provide as many or as few ways of improving how participants were being recruited. In essence, this task provided participants with an opportunity to support evidence-based behavioral research–which adhered to the pledge that they had signed. We then measured the number of suggestions each participant provided, which served as the behavioral measure of commitment.

The instruction made it clear that the participants were under no obligation to either sign the pledge or provide any suggestions to the experimenters. Regardless of their behaviors and the pledging condition to which they were randomly assigned, participants then provided their demographic information and were thanked and excused.

### Results

All participants signed the pledge to help advance behavioral research. However, more than half of the participants (53.8%) did not provide suggestions. This prompted us to conduct a Poisson regression analysis to gain a more detailed understanding of how much people actually helped. We used a Poisson regression analysis because it allows us to preserve the meaningfulness of the zeros in our data and because the dependent variable is a count variable. Table 1 presents the full results of the regression analysis along. As predicted, the pledge-signing manipulation had a significant impact on commitment behavior *χ*^*2*^(2) = 16.59, *p* < .001. Parameter estimation with the signed by hand condition as the reference group indicated that both the checked box (B = -.86, SE = .23, *χ*^*2*^ = 13.92, *p* < .001) and the typed initials condition (B = -.64, SE = .24, *χ*^*2*^ = 6.94, *p* = .008) differed significantly from the handwritten condition. Pairwise comparisons further revealed that those who signed the pledge by hand volunteered more suggestions (*M* = 1.82, *SD* = 2.27) than those who pledged via checking a box (*M* = .77, *SD* = 1.33; *p* < .001, Cohen’s *d* = .56) or typing their initials (*M* = .96, *SD* = 1.26; *p* = .008, Cohen’s *d* = .46). The checked box condition did not significantly differ from the typed name condition (*p* = .45).

**Table 1:** Poisson regression analysis on commitment behavior, Pilot Study 1A.

Variable	B	SE	95% CI
Pledge Condition			
Check Box	-.86***	.23	[-1.31, -.40]
Type Initials	-.64**	.24	[-1.12, -.16]
Hand-signed^a^	-	-	-
Intercept	3.64***	.12	[.35, .85]

*p<.05;

**p<.01;

***p<.001

^a^Hand-signed condition served as the reference group

### Discussion

Results from Pilot Study 1A reveal that although everyone received the same text in the pledge, how they signed it significantly affected whether and how much they helped to further the cause. In short, common forms of e-pledging are indeed less effective at securing commitment than the traditional form of signing pledges by hand. This discrepancy further highlights the importance of bolstering and solidifying e-pledges’ effectiveness as a commitment tool. Pilot Study 1B set out to further understand why this effect occurs.

## Pilot Study 1B: Why conventional e-pledges are ineffective

We conducted an online survey study using the Amazon Mechanical Turk platform (MTurk). The objective of this study is to understand laypeople’s perception of the shortcomings of conventional e-pledges. As these online participants came from the population frequently targeted for online campaigns and e-pledges, we reason that their responses would be a valuable and valid source of information.

### Methods

#### Participants and procedure

Three hundred and one participants recruited from the MTurk online platform completed the survey online (44% female; mean age = 35.10, *SD* = 9.91). We obtained IRB approval from the University of Virginia to conduct this study, with written consent from the participants. After entering their MTurk ID, participants read the definition of slacktivism, which was defined as “a phenomenon in which people pledge support for a cause on social media without following up with actual behaviors that contribute to the cause.” They then responded to two blocks of survey questions in sequence to measure their perceptions of (1) the prevalence and severity of slacktivism and (2) why e-pledges may fail to work. We describe each of the blocks in more detail below. Participants also had the opportunity to provide open-ended comments on slacktivism at the end of the survey. They were not obligated to provide any responses to this question; in our final data, only 96 participants provided any comments, half of which were not related to slacktivism. Therefore, we did not submit the data to any systematic qualitative analysis.

#### Measures of the prevalence and severity of slacktivism

After reading the definition of slacktivism, participants were told that the experimenters would like to learn more about their perception of the prevalence and severity of slacktivism. Participants then responded to three questions: whether they had personally engaged in slacktivism in the previous 6 months (1 = *definitely not* to 5 = *definitely yes*); how prevalent a problem they think slacktivism is (1 = *not at all* to 5 = *very prevalent*); and how serious a toll it takes on society at large (1 = *not at all* to 5 = *a great deal*).

#### Measures of why e-pledges may fail

After completing the block on slacktivism prevalence and severity, participants were then asked to reflect on reasons why e-pledges may be ineffective. We grounded these reasons in past research, which highlight two competing forces that may contribute to slacktivism. Participants first reviewed five reasons that may have contributed to e-pledging’s failure to secure commitment and indicated how much they thought each contributed to slacktivism (using a 5-point scale: 1 = *not at all* to 5 = *very much so*). Two of the reasons focused on the pledger’s reaction to the pledge (*“People feel less accountable to the pledges and petitions they sign online”; “People feel less guilty for breaking online pledges and petitions”*), and three that concerned the pledge itself (“*There are too many online pledges and petitions around”; “People often question the veracity of the online pledges and petitions”;* and “*Online pledges and petitions are less emotionally appealing”*). After rating each of the five reasons, participants then ranked order them from 1 = *most important* to 5 = *least important*.

### Results

#### Severity of the issue

We submitted participants’ ratings of their personal involvement in slacktivism, the prevalence of slacktivism, and the severity of slacktivism to a series of one-sample *t*-tests. Results revealed that all three were significantly more than the midpoint of the response scale (*t*(300) > 5.86, *p* < .001). Most notably, 58.8% of participants indicated that they most likely or had definitely committed slacktivism in the previous 6 months, and 80.1% indicated that it is a prevalent or very prevalent issue. In total, 46.9% of participants agreed that slacktivism is a serious to very serious issue for society.

#### Why e-pledges do not work

**Fig 1:**
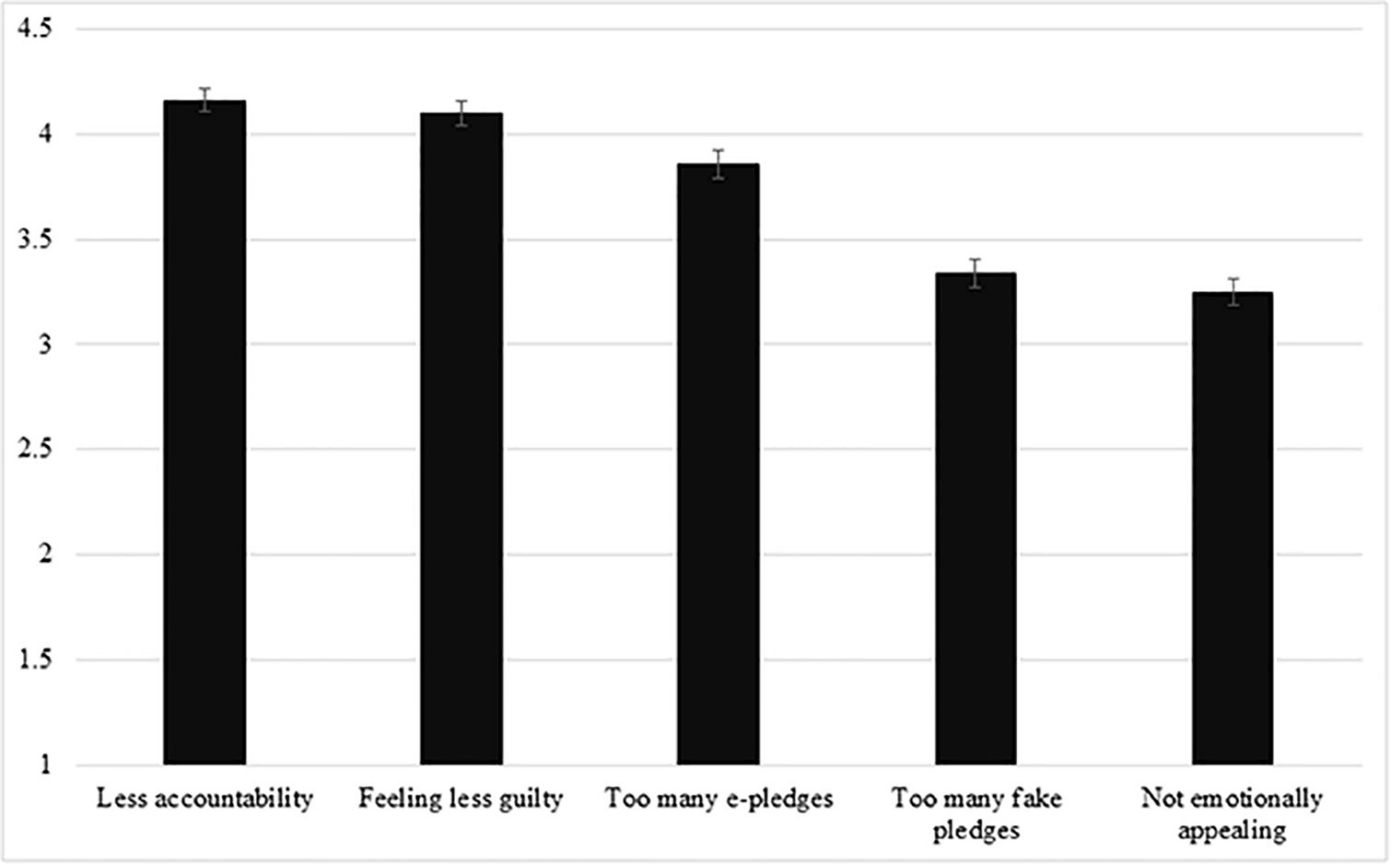
Ratings of factors that contributed to slacktivism, Pilot Study 1B.

Because participants provided individual ratings for each of the five reasons, we employed a repeated measures ANOVA on the rating data to test equality of means of the five reasons. As all of the participants rated the same five statements, a repeated measures ANOVA would allow us to investigate whether there were overall systematic differences across how people responded to those statements. Results revealed a significant difference in lay perception of what contributes to slacktivism (*F*(4, 1200) = 60.74, *p* < .001, *η*^*2*^ = .16; Fig 1). The overall significant effect further granted us the ability to assess pairwise differences between the statements. Subsequent pairwise comparisons showed that “less-accountability” (*M* = 4.16, *SD* = .96) and “less guilt” (*M* = 4.10, *SD* = 1.00) were significantly different from the remaining three reasons (*p*_*s*_ < .001). However, these two reasons were not perceived to be contributing differently (*p* = .34).

We then examined forced ranking data. A Friedman nonparametric test demonstrated that there were overall differences in the rankings of why people thought e-pledgers shirk (*χ*^*2*^ (4) = 327.87, *p* < .001). Participants’ rankings identified “lacking accountability” (*M* = 1.95) and “feeling less guilty” (*M* = 2.35) as the two highest rated reasons for why e-pledgers shirk. The remaining reasons, in order of rank, were “too many pledges available” (*M* = 3.16), “online pledges are often fake” (*M* = 3.72), and “not as emotionally appealing” (*M* = 3.82). Friedman follow-up tests revealed that “less accountability” was ranked to be significantly more important than “feeling less guilty (*p* < .001), and each were ranked as significantly more important than the third-ranked item, “too many pledges available” (*p* < .001).

### Discussion

This exploratory study yielded several insights. First, it provided empirical support for the prevalence of slacktivism. Second, not only do people recognize the severity of the issue, they also acknowledge that they themselves have been culprits. This discrepancy between willingness to pledge and subsequent commitment behavior further accentuates the importance of improving e-pledges’ effectiveness as a commitment tool.

More importantly, the pilot study provided directions for how we could improve conventional e-pledges. Our results identified (1) weak accountability and (2) lack of emotional investment as major contributors to slacktivism; both reasons rest more on the method of e-pledging itself than on the pledges in general. These findings suggest that to strengthen e-pledges and curb slacktivism, we would need to think about ways to boost accountability and heighten the sense of emotional consequences of the pledge. These findings enabled us to design potential interventions that may be more effective.

## Study 1: E-pledging to volunteer in the field setting

Results from our two pilot studies served as a springboard for potential ways to improve e-pledges’ effectiveness. When developing interventions, it is essential to keep any constraints and boundaries in mind. Furthermore, the intervention should preserve the current system’s strengths while improving it. We aimed to preserve two parameters. First, two strengths of e-pledges are their efficiency and ease of administering [18]. Any modification, therefore, should be neither cumbersome for pledgers nor difficult for campaigns to disseminate. Similarly, the proposed method should be as generalizable as possible across different causes; otherwise, too much tailoring to the cause or specific campaign may limit broad usage.

With these caveats in mind, we focused our attention on how to boost psychological accountability and a sense of normative pressure when people sign an e-pledge. We reasoned that one way to improve an e-pledge’s commitment power is to raise the sense of public self-awareness as people e-pledge. Public self-awareness is the state in which people focus on the impressions they make on others, based on their behavior and appearance [9]. In such a state, people observe their own behaviors from the vantage point of real others and seek social approval [19–21]. In turn, public self-awareness induces greater accountability and leads people to act in line with perceived social norms and personal standards.

Notably for our purpose, public self-awareness can be activated using different accountability cues [21–24]. For instance, a classic example of an accountability cue is the mirror manipulation [23]: placing an individual in front of a mirror so that their image was visible throughout the experiment served to heighten participants’ self-awareness. Likewise, the presence of a camera can induce public self-awareness [25]. For instance, being in front of a webcam with other people who, potentially, are watching the webcam feed can also heighten accountability [20].

We propose that asking people to pledge with their own name plus the names of someone important to them would be a two-pronged approach to creating such an accountability cue. First, this self–other e-pledge could heighten psychological accountability by introducing a virtual audience as people e-pledge [20, 26–28]. The familiar nature of the other person’s name would also lead e-pledgers to perceive the “virtual audience” as more legitimate: People respond better when they envisage having to explain their actions to a friend (a legitimate audience) than to a random stranger [an illegitimate stranger; 29–31]. Furthermore, the sense of social surveillance, achieved through real or imagined presence of others during acts, has been shown to fuel public self-awareness [20, 32–33]. In turn, we predict that a heightened sense of public self-awareness should lead to more socially desirable behavior [20, 34].

Second, this self–other e-pledge could emphasize core personal standards and normative expectations, because the introspective process by which people contemplate who is important to them could draw attention to the self [32, 35]. When people are more focused on the self, they become more attuned to important personal standards and feel pressured to act in line with their core identity [36]. Because people have an inherent desire to see themselves as consistent across behaviors, this activated sense of self compels them to comply with the behaviors targeted by the pledge [12]; several empirical studies have documented the mobilizing power of self-focus in securing prosocial behavior [21], which lend indirect support to our prediction.

Taken together, we set out to test a novel e-pledging method aimed at increasing public self-awareness: self–other pledging. This method requires that e-pledgers pledge not only with their own name but also with the name of someone important to them and whom they respect. It is also important to note that although pledgers are using two names to pledge their support, they are making the pledge on their own behalf and not the other person’s. In effect, e-pledgers are dedicating their effort to the other person they included in the pledge.

To test the effectiveness of our proposed intervention in a context with high ecological validity, we conducted a field experiment in collaboration with a university-affiliated student volunteer center that sponsors various programs in the community. One of the major challenges most nonprofit organizations face nowadays is high volunteer attrition [37]. Because these organizations greatly depend on volunteers to function, securing volunteer commitment is critical. Our goal was to investigate whether our proposed e-pledge intervention would function better at securing volunteering commitment than conventional methods.

At the beginning of the fall semester, students who had registered for one of the center’s volunteering programs were asked to take an e-pledge of commitment. They were randomly assigned to one of three methods of e-pledging: two conventional methods (signing with their name or by clicking on “Like”) and our proposed method (combined self–other names). We predicted that at the end of the semester (three months later), those who pledged with combined names would volunteer significantly more hours than those who pledged with either of the conventional methods.

### Methods

#### Participants recruitment process

At the time of data collection, the center sponsored 27 volunteering programs around the community, such as youth mentoring, tax services, housing improvement, and medical services. Each of these programs was headed by a program director, who oversaw the management of their volunteers throughout the duration of the semester. We worked directly with program directors at the volunteer center because of their proximity to the organization process. Eighteen program directors agreed to work with us. During the time of our data collection, the 18 program directors were tasked to manage 140 volunteers in total. Per our arrangement with the program directors, we were permitted to embed an e-pledge at the end of a standardized and mandatory online survey that all volunteers receive at the beginning of the semester. The online survey was administered by the program directors [38]. We were also given access to the timecards at the end of the semester, along with basic demographic information of the volunteers (i.e., age, year at school, tenure with the program, and gender). We were not permitted to contact the volunteers during the course of the semester or after the semester had ended. We obtained IRB approval from the University of Virginia to conduct this study, with written consent from the participants.

At the end of the semester, eight volunteers terminated their involvements with the volunteering programs all together (four from the Like condition, one from the initials condition, and three from the combined self-other initials condition; see Online Supplemental Material S1 Table for more detail). Therefore, our final data set comprised of 132 volunteers across 18 volunteering programs (77% female; mean age = 19.51, *SD* = 1.14; tenure with program = 1.76 semesters, *SD* = .87). We did not have access to the reasons why those eight volunteers terminated their involvements, nor did we have access to the number of hours they worked prior to terminating their involvement with the programs. We conducted a Chi-square analysis to assess whether attrition was related to experimental condition. Results revealed that there was no significant relationship between condition and attrition (*χ*^*2*^(2) = 1.79, *p* = .40). We also conducted a correlation analysis to verify that the random assignment to experimental conditions and volunteering programs were not correlated. The Pearson Correlation Coefficient between the volunteering program and experimental condition is 0.014 (*p*-value = .865). This result suggests that both variables are not correlated with each other.

#### Pledge manipulation

The program directors informed us that it is customary for all volunteers to complete an online survey at the beginning of the semester that asked volunteers to provide basic demographic information, such as age and gender, and their proposed schedule. This provided us with the opportunity to institute an e-pledge. At the end of the survey that they would normally complete, volunteers were asked to pledge that they would honor their commitment throughout the 3-month period. All of the participants read the same content of the pledge:

*“On my honor*, *I pledge to uphold the values of a good volunteer*. *I will consistently attend my volunteer shifts on time*, *I will do my best to work enthusiastically and maintain a positive attitude*, *and I am committed to seeing my work through until the end of the semester*.”

Although the pledge’s content was identical, we randomly assigned volunteers to one of three e-pledge formats: two conventional methods (clicking on “Like” or typing their initials) and the combined self-other initials e-pledge. We randomized participants to different e-pledging conditions regardless of the program. Following prior research, we asked participants to use initials instead of their full names in order to protect their identity and maintain anonymity [see 16].

Those in the **“Like” e-pledge condition** read the above statement and were asked to “*Please sign the pledge by clicking on the “Like” symbol*”.

Those in the **“Self-initials” e-pledge condition** read the pledge above and were asked to “*Please sign the pledge by entering your initials*.”

Those in the proposed intervention–the “**Combined self-other initials” e-pledge condition** read the pledge above and then were asked to “*Think of a person who is extremely important to you and helped you to become the person you are today*, *then sign the pledge with both your own initials plus the initials of that person*.”

All of the volunteers opted to take the pledge. The extent of our involvement with the programs or the volunteers ended after that initial online survey. Volunteers went about their routine and did not receive any reminders or follow-up surveys from us. At the end of the semester, we obtained the logs of volunteer hours from the program directors; the logs tracked volunteers’ actual work hours across the semester, which served as our dependent variable.

#### Control variables

We consulted with program directors to understand the demographic variables that have systematically affected volunteering hours in the past. Based on that information, we controlled for each volunteer’s age, sex, and any previous volunteer experience with the nonprofit organization. These information were part of the mandatory survey that volunteers filled out at the beginning of the semester.

#### Analytical approach

We analyzed the data in two ways. We first used path modeling to analyze whether the e-pledge manipulation had a significant impact on volunteering hours. Because (a) different programs may require different levels of involvement, with some being more time-intensive than others (e.g., daily after-school tutoring versus cleaning up a river bank on weekends), and (b) the e-pledging conditions were randomly assigned to volunteers regardless of the program, volunteering hours in our experiment were not independent across programs. Therefore, we modeled the programs’ nonindependence using the cluster command in Mplus 7 [39], which adjusts standard errors for nested data structures.

We also conducted Poisson regression analyses to further understand whether those who pledged with combined initials would volunteer significantly more hours than those who pledged using conventional methods (i.e., “Like” clicking and name initials typing), above and beyond the control variables. We adopted a Poisson regression analysis because the dependent variable is a count variable.

### Results

**Fig 2:**
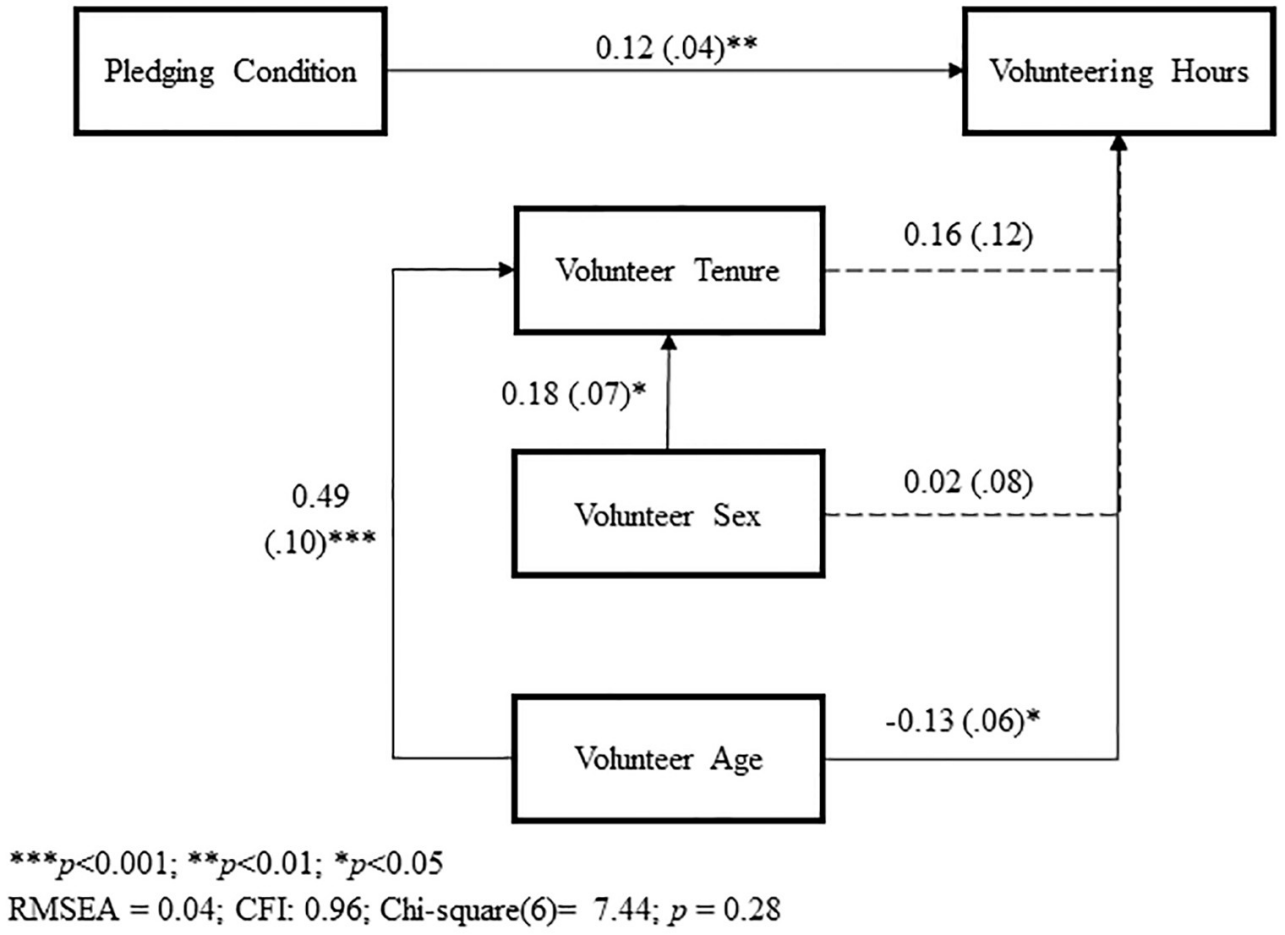
Path model analysis, Study 1.

We conducted a single-indicator nested modeling analysis clustered on programs, controlling for volunteer’s age, sex, and their previous volunteer experience with the nonprofit organization. Fig 2 presents the path coefficient. We first examine the overall model to determine whether it confirmed that the pledging condition had a significant effect on hours volunteered. This analysis revealed that model fit indices satisfied the goodness of fit standard [CFI = 1.00, RMSEA = 0.00, χ^2^(6) = 5.53, *p* = .47; Fig 2; 40]. We then focused on the impact of the intervention. As predicted, those who e-pledged with combined initials volunteered significantly more hours throughout the semester than the those who pledged with conventional methods (B = .11, *SE* = .04, *p* = .006; CFI = 1.00, RMSEA = 0.00, χ^2^(6) = 5.05, *p* = .53).

Poisson regression analysis shed more light on the self-other initials effect (see Online Supplemental Material S2 Table). Model 1 included only the experimental conditions. Model 2 showed that the effect strengthen after including program as a control variable. Model 3 included all remaining control variables–volunteers’ gender, age, and tenure. Results showed that, after controlling for the control variables, volunteers in both “Like” and “self-initials” conditions volunteered significantly fewer hours than those who pledged via “self-other initials.” Volunteers who took the “like” e-pledge (*M* = 7.29 hrs, *SD* = 4.93) worked significantly fewer hours than those who took the “self-other” e-pledge (*M* = 9.09 hrs, *SD* = 5.45; B = -.56, *SE* = .25, *p* = .02). Those who pledged with their self-initials (*M* = 8.12 hrs, *SD* = 4.80) also worked fewer hours than those e-pledged with “self-other” initials (B = -54, *SE* = .27, *p* = .05). This indicates the robustness of the self-other initials effect.

### Discussion

Study 1 provides the first validation of our self-other initials intervention. By asking volunteers to pledge with both their own initials and another important other’s, they volunteered 24.69% longer over a span of 3 months than those who pledged with the “Like” button, and 11.94% longer than those who pledged with self-initials only.

This field study’s longitudinal design also boosts the external validity of the effect. These findings, while supportive of our predictions with strong external validity, are less controlled due to the nature of field studies. For instance, the number of volunteers who participated in this study was outside of our control. Additionally, as a compromise we had to make to gain entry to the organization, we agreed to make the experimental design as nonintrusive as possible. Hence, the only volunteering information we were able to retrieve was the number of hours. To address these concerns and enhance the causal inference of our findings, we conducted Studies 2 and 3 with better experimental control to complement our initial findings.

More importantly, results from Study 1 reveal that although everyone received the same pledge, how they signed affected whether and how much they helped in a tangible manner to further the cause. Yet, results from Study 1 did not speak to why pledging with the name of someone important to them made a significant difference in behavior. On the one hand, participants might have instinctively dedicated their effort to the person they named in their pledge. On the other hand, simply recalling a person might have been sufficient to improve commitment. Study 2 set out to refine the intervention by explicitly asking participants to dedicate their efforts to that person who is very important to them.

## Study 2: E-pledging to act

Study 2 strived to achieve four goals: First, we aimed to gain greater control and reduce the noise that is inherent in field experiments. To do so, we created an experimental design in which participants had the opportunity to e-pledge their commitment to the same cause, via different e-pledging methods. We viewed this as a more conservative test of the e-pledge intervention, because people were all pledging to the same cause and they were randomly assigned to the various e-pledging methods.

Second, we set out to better understand why pledging with people’s own initials plus that of someone important and impactful to them led to greater commitment in Study 1. To do so, we created two combined-initials condition. The first asked participants to think of the name of someone in their life who comes to mind, then take the pledge with their own initials plus the initials of that person. We termed this the *self & top-of-mind condition*. The second asked participants to think of someone in their life that they would like to dedicate their efforts to, and then take the pledge with their own initials plus the initials of that person. We term this the *self-dedication condition*. By comparing the commitment effects of these two different combined-initials conditions, and contrasting them to the commitment of those who pledged with just their own initials, we can gain a deeper understanding of the underlying psychological mechanism that led participants to be more committed in a given cause.

Third, we aimed to rule out pledging effort as an important potential alternative explanation; that is, the intervention required that e-pledgers exert more effort than participants using conventional methods for e-pledges. It is conceivable that the amount of effort required to e-pledge may have driven the positive effect on commitment. To control for this, we measured how long it took participants to take the pledge. We then examined if there were any systematic differences across conditions, and, whether that would explain the predicted difference in commitment.

Fourth, while it was described as optional, everyone in Study 1 signed the pledge. In Study 2, we made it even more explicit that signing the pledge was optional, in an effort to minimize any potential demand effect. We also tracked whether people opted out of signing the pledge across different e-pledge conditions.

### Methods

#### Participants and design

We aimed to recruit 330 participants from MTurk with an IP address based in the US in exchange for $0.81. At the end of the predetermined data collection period, we yielded 329 valid responses (mean age = 35.35, *SD* = 11.21; 41.77% female). We obtained IRB approval from the University of Virginia to conduct this study, with written consent from the participants. Participants were randomly assigned to one of three conditions: (a) pledging by their own initials (self-initials), (b) pledging with their own initials plus the initials of a person in their life who comes to mind (self & top-of-mind), and (c) pledging with their initials plus the initials of someone to whom they would like to dedicate their efforts to (self-dedication initials).

#### E-pledge manipulation

Participants first read a two-paragraph statement about child hunger in the U.S. For instance, they learned that one in five children in the U.S. lack proper nutrition and access to food at some point during the year [41]. Participants then read the following pledge:

*Any action you take will work toward the same goal—to confront child hunger and give our future generations the nourishment they need to thrive*. *Let us join efforts and commit to help raise awareness for the cause in the next few weeks*.

Participants were then given the option of pledging their support in one of three ways: self-initials, self & top-of-mind, or self-dedication initials.

Those in the **“Self-initials” e-pledge condition** read the pledge above and were asked to “*Please sign the pledge by entering your initials*.”

Those in the proposed intervention–the “**Self & Top-of-Mind” e-pledge condition** read the pledge above and then were asked to “*Think of someone who comes to mind*, *then sign the pledge with both your own initials plus the initials of that person*.”

Those in the proposed intervention–the “**Self-Dedication initials” e-pledge condition** read the pledge above and then were asked to “*Think of a person who is extremely important to you and helped you to become the person you are today*, *then dedicate the pledge that person with both your own initials plus the initials of that person*.”

#### Commitment behavior

After signing the pledge, participants were given the opportunity to list the concrete steps they would take to help end child hunger. The instruction also stressed that they could list as many or as few steps as they wished. It was also made clear that the participants’ compensation for the experiment was not tied to the number of commitment actions they would take.

All of the participants provided their demographic information at the end of the survey and were paid the next day.

### Results

#### Opt-out rate

We began by examining how many participants chose not to pledge as a function of the three e-pledging conditions. Twenty-seven participants opted out of signing the pledge. Results revealed that whereas 11.57% of those in the self-initials (n = 14) and 10.67% in the self & top-of-mind conditions chose not to pledge (n = 11), only 1.90% of the participants in the self-dedication condition declined to pledge (n = 2; *χ*^*2*^(2) = 8.18, *p* = .01). The significantly lower attrition rate demonstrated that people were not deterred by the self-dedication condition. The results remained significant if we were to exclude participants who did not sign the pledge of the data.

#### Commitment behavior

Results from one-way ANOVA revealed that the pledging manipulation affected participants’ commitment to the cause, *F*(2, 326) = 5.01, *p* = .007, partial *η*^*2*^ = .03. Post hoc analysis revealed that those in the self-dedication initials condition (*M* = 3.33, *SD* = 2.63) generated significantly more concrete actions that they would take than those in the self-initials (*M* = 2.73, *SD* = 1.71; *p* = .03; Cohen’s *d* = .27) or the self & top-of-mind (*M* = 2.43, *SD* = 1.83; *p* = .002; Cohen’s *d* = .39) conditions.

We also examined whether it would take longer to sign the pledge across the three conditions, and whether this would partially explain the commitment effect. One-way ANOVA revealed that the pledging manipulation did indeed affect how long it took to complete the pledge, *F*(2, 326) = 8.13, *p <* .001, partial *η*^*2*^ = .04. Post hoc analysis revealed that those in the self-dedication (*M* = 21.48 s, *SD* = 34.00) condition took significantly longer to pledge than those in the self-initials (*M* = 9.55 s, *SD* = 11.36; *p <* .001) and those in the self & top-of-mind conditions (*M* = 15.31 s, *SD* = 13.78; *p* = .04). Those in the self & top-of-mind condition also took longer to pledge than the self-initials condition (*p* = .05).

To assess whether the effort it took to take the pledge would explain the commitment effect, we conducted a MANOVA analysis with pledging effort as a covariate. The results revealed that the pledging commitment remained significant and strong (*F*(2, 325) = 5.17, *p* = .006, partial *η*^*2*^ = .03), despite including pledging effort as a covariate. Pledging effort did not have a significant effect on commitment, *F*(1, 325) = .34, *p* = .55. Together, these results suggest that pledging effort alone may not have been sufficient to explain why the self-dedication initials would deepen people’s commitment to the cause they pledged to support.

### Discussion

Results from Study 2 extend our understanding in three ways. First, the findings provided a deeper understanding of the effectiveness of the proposed intervention. It is worth noting that the effect remained robust even when all of the participants read about the same nonprofit organization and received the same pledge: the only difference was how they signed the pledge. Second, we showed that the self–dedication initials’ effect on commitment was independent of the effort exerted to sign the pledge. This is supported both by how long it took to sign the pledge and by including a condition in which participants pledged with their own initials plus the initials of someone who came to mind. Third, results on the differential opting-out rate further demonstrate that the self-dedication pledging method did not systematically deter people from engaging in the pledge. To the extent that the intervention could be practically implemented in the field, it is reassuring to see that this method may have encouraged, rather than discouraged, potential pledgers to sign the pledge.

## Study 3: E-pledging to commit

Study 3 had two main goals: First, we set out to replicate the results from Study 2 in a more controlled setting. Second, we aimed to rule out pledging effort as an important potential alternative explanation in a different way than in Study 2. To do so, we included a condition in which participants were asked to include two computer-generated letters in their pledge. These computer-generated random letters would increase the effort it took for participants to e-pledge without increasing self-focus, unlike the proposed intervention. Same as in Study 2, we tracked whether participants opted out of signing the pledge across different e-pledge conditions.

### Methods

#### Participants and design

We aimed to recruit 360 participants from MTurk with IP addresses based in the US in exchange for $0.76. At the end of the predetermined data collection period, we received 348 complete responses (mean age = 35.18, *SD* = 10.34; 38.79% female). We obtained IRB approval from the University of Virginia to conduct this study, with written consent from the participants. Participants were randomly assigned to one of three conditions: (1) pledging with their own initials, (2) pledging with their own initials plus two random letters, and (3) pledging with their initials plus the initials of someone important to them.

#### E-pledge manipulation

Participants first read a two-paragraph description of a U.S. nonprofit organization, the Boys and Girls Club of America (BGCA). They read about BGCA’s functions and the various ways people could get involved in improving a child’s life. Participants then read the following pledge:

*Any action you take will work toward the same goal—to strengthen and empower children in need*. *Join the Boys and Girls Clubs of America campaign by committing yourself to help raise awareness for the foundation in the next few weeks*.

Participants were then given the option of pledging their support in one of the three ways: self-initials, self-initials plus initials of someone very important to them (self-dedication initials), or self-initials plus two randomly generated letters (self & random letters). The phrasing of the self-initials and the self-dedication initials pledges were the same as in Study 2. Those in the self & random letters condition read “*Please sign the pledge by entering your initials plus the two letters shown below*.” These two letters were randomly generated by the computer program for each participant. For instance, someone with the initials EC would pledge with her initials plus two letters that were randomly generated by the program (i.e., ECNM).

#### Commitment

Participants responded to three questions that captured their commitment to the cause: “I will tell other people of the Boys and Girls Club”; “I feel very committed to missions of the Boys and Girls Club”; and “I feel very connected to the children being served by the Boys and Girls Club.” We averaged responses to form the commitment scale (α = .83).

All of the participants provided their demographic information at the end of the survey and were paid the next day.

### Results

#### Opt-out rate

We began by examining how many participants chose not to pledge as a function of the three e-pledging conditions. Results revealed that whereas 10.74% of those in the self-initials (n = 13) and 13.15% of the self & random letters conditions (n = 15) chose not to pledge, only 3.53% of the participants in the self–dedication initials condition (n = 4) declined to pledge (*χ*^*2*^(2) = 6.82, *p* = .03). The significantly lower attrition rate demonstrated that people were not deterred by the self-dedication initials condition. Results remained significant if we were to include excluded participants who did not sign the pledge.

#### Commitment

Results from one-way ANOVA revealed that the pledging manipulation affected participants’ commitment to the cause, *F*(2, 345) = 6.48, *p* = .002, partial *η*^*2*^ = .03. Post hoc analysis revealed that those in the self-dedication initials condition (*M* = 3.47, *SD* = 1.06) were significantly more committed to the cause than those in the self-initials (*M* = 2.99, *SD* = 1.03; *p* = .001; Cohen’s *d* = .45) or self & random letters (*M* = 3.09, *SD* = 1.09; *p* = .008; Cohen’s *d* = .35) conditions.

We also examined whether it would take longer to sign the pledge across the three conditions, and whether this would partially explain the commitment effect. One-way ANOVA revealed that the pledging manipulation did indeed affect how long it took to complete the pledge, *F*(2, 345) = 6.95, *p* = .001, partial *η*^*2*^ = .03. Post hoc analysis revealed that those in the self-dedication initials (*M* = 19.18 s, *SD* = 22.48) and self & random letters (*M* = 15.76 s, *SD* = 30.02) conditions took significantly longer to pledge than those in the self-initials condition (*M* = 8.57 s, *SD* = 10.30; *p*_*s*_ *<* .01 for both conditions). It did not take significantly longer for people to pledge using self-other or self & random letters (*p* = .24).

To assess whether the effort it took to take the pledge would explain the commitment effect, we conducted a MANOVA analysis with effort as a covariate. The results revealed that the pledging commitment remained significant and strong (*F*(2, 344) = 5.31, *p* = .005, partial *η*^*2*^ = .03), even with effort as a covariate. Importantly, effort did not have a significant effect on commitment, *F*(1, 344) = 3.17, *p* = .07. Together, these results suggest that effort alone may not have been sufficient to explain why the self-dedication initials would deepen people’s commitment to the cause they pledged to support.

## General discussion

Comedian Seth Meyers once said, “If you make a Facebook page we will ‘like’ it—it’s the least we can do. But it’s also the most we can do.” While meant to be satirical, his statement largely reflects a reality of social media: Conventional e-pledges and online campaigns are the new and increasingly ubiquitous reality for nonprofit organizations and advocacy groups [42–43], yet their convenience and broad reach may be offset by their ineffectiveness for securing committed behaviors. With the goal to improve this dynamic, we conducted two pilots and three experiments to empirically examine whether a novel and simple e-pledge intervention—self–other initials—is more effective for securing commitment behavior than other types of common e-pledges. To that point, we refined the pledge by asking participants to explicitly dedicate their thoughts to that other person, and ruled out effort, novelty, and social interaction mindset as alternative explanations. These results provide a new and enhanced method that may enable organizations to capitalize on the benefits of e-pledging without compromising on social media’s mass outreach effect.

Notably, the positive link between self–other e-pledging and volunteering commitment over a span of 3 months further enhances confidence in the external and ecological validity of our findings (Study 1). The self–other e-pledge’s ability to secure commitment behavior through explicit dedication also highlights the importance of enforcing accountability cues in the e-pledging process (Studies 2 and 3). Together, our findings aimed to explain whether and how a common and increasingly prevalent method for obtaining commitment—e-pledging—can effectively be strengthened to secure a wider range of prosocial behaviors that are otherwise difficult to motivate [44].

It is important to point out that regardless of the e-pledge condition participants were assigned to, virtually all of them self-elected to take the pledge in Study 1, and a significant portion of the participants did so in Studies 2 and 3. Nevertheless, the actual commitment behavior varied significantly across e-pledging conditions. The discrepancy between signing the pledge and taking action provides further empirical evidence for slacktivism, consistent with survey responses from our Pilot Study 1B. This also suggests that participants’ recognition of the importance of these social causes and that at least at the time of pledging, they may have intended to honor their pledges. Therefore, the effectiveness of our intervention was unlikely to be a result of participants’ insincerity or an experimental demand effect. Even so, we consistently showed that small yet carefully planned interventions–such as how people pledged—can greatly impact their committing behaviors [see 45]. Specifically, compared to those who pledged with a “Like” symbol—a widely used method of e-pledging across social media—those who pledged with combined initials volunteered 24.69% longer across the span of 3 months (Study 1).

### Theoretical contributions and frameworks for future research

Our research makes several theoretical contributions and initiates new research directions. First of all, our findings shed light on laypersons perspective of slacktivism, speak to and integrate theoretical perspectives on social influence and public self-awareness, and provide support for a different and more effective method of e-pledging. These results form the basis for fruitful research endeavors. We describe the linkage to previous literature and outline possible future directions below.

Results from the pilot studies showed that laypeople acknowledged the e-pledging process as the main contributor to slacktivism, citing lack of accountability and consequence as major factors. Building on these results and drawing from public self-awareness research, we predicted that an effective intervention would incorporate an accountability cue in the moment people signed the pledge in the virtual environment. In a sense, our proposed intervention embedded a virtual mirror as people signed the pledge. Results from Studies 1–3 provide empirical support for our proposed intervention. Namely, the self-other intervention required participants to generate their own standard. Research on public self-awareness theory postulates that people would then measure their subsequent behavior by the standard they have just created. This heightened sense of self-regard would therefore compel them to follow through with the e-pledge.

While we have ruled out several possible alternative explanations, we acknowledge that others may exist as well. One such explanation might be that the intervention evoked a “significant-other transference.” Namely, contextual cues not only work to activate a sense of self, but different cues can affect which version of the self is activated [46, 47]. For instance, cues in a family setting can elicit affective, motivational, and behavioral responses associated with one’s “family-oriented self,” and this activation of the relational self can spill over to a different context [46, 48–49]. Therefore, it is possible that e-pledgers became more committed because it activated the relational self. The positivity and intimacy derived from transference can boost the pledger’s likelihood of carrying out the pledged behavior. Future research may shed more light in this direction by investigating this and related alternative explanations.

Our results also have point to broader questions for research on conventional e-pledges and slacktivism. Prior research has demonstrated that people often divide a superordinate goal into incremental subgoals. How people interpret these subgoals, in turn, has direct implications for goal pursuit. Specifically, when people interpret their success in fulfilling a subgoal as a possible substitute for the superordinate goal, they are less likely to pursue the superordinate goal [50]. Applying that to the current context, one reason that contributes to slacktivism could be due to pledgers feeling that they have already taken steps to support the cause. A more comprehensive understanding of the interplay between these forces would be a promising direction for future research.

Along similar lines, it is possible that people may simply disengage from commitment and responsibility after e-pledging; research on moral licensing suggests this potentially troublesome consequence. Namely, e-pledgers could feel that they have already obtained the feel-good “moral credits” just by clicking on the pledge, and therefore free themselves to engage in opposite behavior away from the commitment [51]. Beyond this possibility, we encourage future research to examine potential negative consequences that may result from these externally induced psychological barriers.

### Practical implications

There are several ways our research informs organizations that seek to promote their causes. We have focused on the self–other pledge as one way to awaken and reinforce accountability. Organizations may want to further tailor their pledging prompt by more explicitly asking people to generate names of others who are relevant in that particular domain. For example, St. Jude’s campaign could ask e-pledgers to dedicate their efforts to others who had courageously battled an illness, either personally or in the signer’s role as a caregiver. Similarly, the American Red Cross could suggest that the “other” be someone who generously and faithfully provides assistant to people in need in their communities. By tailoring these messages to the essence of their cause, organizations may be even better served by this intervention.

Our findings also highlight a more cost-effective method for reaching a broad volunteer base and keeping them engaged. Currently, nonprofit organizations often send reminders of the recipient’s prior commitment. However, not only is this practice costly, but reminding people about previous pledges may also backfire. Namely, research has demonstrated that people sometimes react to reminders of past commitment failures by veering away from their original moral standards [52–54]. If the e-pledger needs to spend a lot of effort to make up for the failure (i.e., physical distance is far or time commitment is high), then reminding them of the e-pledge they made may inadvertently push them further away and motivate them to disengage. This puts organizations in a difficult position. Our research suggests that it may be more effective to incorporate an intervention at the moment of e-pledging rather than waiting until the behavior has already occurred [see 22 and 24]. In short, our research provides a more cost-efficient method for nonprofit organizations to secure long-term commitment.

This research also has broad societal implications. In 2019, UC Berkeley lost its #2 *U*.*S*. *News* public university ranking because it provided data on the number of alumni who pledged to donate rather than the actual donation rate [55]. In an ideal world, the number of alumni who pledge to donate would be equal to the number of actual donations. In reality, the discrepancy had a consequential impact on the university, with unknown future ramifications. By systematically investigating the psychological mechanism that can strengthen people’s commitment to the e-pledges they make, and by identifying easily administered e-pledge interventions like the one outlined in this research, we envision this line of work potentially empowers nonprofit organizations to further their causes and secure long-term committed support.

## Conclusion

Our findings have high practical importance for motivating prosocial behaviors—i.e., behaviors that cannot be coerced or stipulated, yet are crucial to a society’s well-being. By identifying a simple, low-cost intervention, our findings suggest ways for nonprofit and advocacy groups to benefit from the convenience and efficiency e-pledges offer without compromising actual and long-term commitment. As social media and web-based technologies continue to redefine how people interact with the world, our findings move us one step closer to a broader understanding of how to transform a simple virtual acknowledgement into deeper commitment—and, ideally, action.

## Supporting information

S1 Table Percentage of volunteers by e-pledge condition per program in Study 1. (DOCX)

S2 Table Poisson regression analysis on hours volunteered. (DOCX)

S1 Data Online supplemental materials: Experimental materials. (DOCX)
