# Supplementary material for: From slacktivism to activism: Improving the commitment power of e-pledges for prosocial causes
Source: PLoS One. 2020 Apr 29;15(4):e0231314. doi: 10.1371/journal.pone.0231314 (PMC7190098; doi:10.1371/journal.pone.0231314)
Supplement: S1 Table — (DOCX) [file pone.0231314.s001.docx]

**Online Supplemental Material**

**S1 Table:** Percentage of Volunteers by e-pledge Condition per program in Study 1

|  | Conditions | | |  |
| --- | --- | --- | --- | --- |
| Volunteering Programs | Like | Initial | Self-other initials | Total N |
| 1 | 60% (50%) | 20% (25%) | 20% (25%) | 5 (4) |
| 2 | 67% | 30% | 0% | 3 |
| 3 | 50% | 25% | 25% | 4 |
| 4 | 0% | 50% | 50% | 2 |
| 5 | 0% | 100% | 0% | 1 |
| 6 | 0% (0%) | 100% (0%) | 0% (0%) | 1 (0) |
| 7 | 55% (62.5%) | 27.8% (31.3%) | 17.2% (6.3%) | 18 (16) |
| 8 | 50% (50%) | 32.4% (34.3%) | 17.7% (15.6%) | 34 (32) |
| 9 | 50% | 25% | 25% | 4 |
| 10 | 47.4% (44.4%) | 26.3% (27.7%) | 26.3% (27.7%) | 19 (18) |
| 11 | 33.3% | 33.3% | 33.3% | 3 |
| 12 | 50.0% | 10.0% | 40.0% | 10 |
| 13 | 28.6% | 42.9% | 28.6% | 7 |
| 14 | 50.0% | 25.0% | 25.0% | 8 |
| 15 | 100.0% | 0.0% | 0.0% | 3 |
| 16 | 50.0% | 20.0% | 30.0% | 10 |
| 17 | 75.0% | 0.0% | 25.0% | 4 |
| 18 | 50.0%  (33.3%) | 25.0%  (33.3%) | 25.0%  (33.3%) | 4 (3) |
| Total % | 50%  (50%) | 28.6%  (29.5%) | 21.4%  (20.4%) | 140  (132) |
