# Supplementary material for: From slacktivism to activism: Improving the commitment power of e-pledges for prosocial causes
Source: PLoS One. 2020 Apr 29;15(4):e0231314. doi: 10.1371/journal.pone.0231314 (PMC7190098; doi:10.1371/journal.pone.0231314)
Supplement: S2 Table — (DOCX) [file pone.0231314.s002.docx]

**S2 Table:** **Poisson Regression Analysis on Hours Volunteered**

| **Variable** | **B** | **SE** | **95% CI** | | **B** | | **SE** | | **95% CI** | | **B** | | **SE** | | **95% CI** | |  |
| --- | --- | --- | --- | --- | --- | --- | --- | --- | --- | --- | --- | --- | --- | --- | --- | --- | --- |
| E-Pledge Condition |  |  | |  | |  | |  | |  | |  | |  | |  | |
| Like | -.31** | .09 | | [-.48, -.13] | | -.59* | | .24 | | [-1.07, -.10] | | -.56* | | .25 | | [-1.06, -.06] | |
| Initials | -.15 | .09 | | [-.32, .04] | | -.52* | | .26 | | [-1.04, .005] | | -.54* | | .27 | | [-1.08, .002] | |
| Self-Other Initials | - | - | | - | | - | | - | | - | | - | | - | | - | |
| Program |  |  | |  | | .001 | | .01 | | [-.03, .03] | | .002 | | .01 | | [-.03, .03] | |
| Age |  |  | |  | |  | |  | |  | | -.10** | | .03 | | [-.17, -.02] | |
| Gender |  |  | |  | |  | |  | |  | | .21* | | .09 | | [.02, .40] | |
| Tenure |  |  | |  | |  | |  | |  | | .17*** | | .04 | | [.08, .26] | |
| Intercept | 2.16*** | .07 | | [2.01, 2.30] | | 2.14*** | | .19 | | [1.75, 2.53] | | 3.64*** | | .74 | | [2.17, 5.11] | |

**p*<.05; ***p*<.01; ****p*<.001

Model 2: Self-Other initials served as the reference group in the e-Pledge condition.
