# Supplementary material for: From slacktivism to activism: Improving the commitment power of e-pledges for prosocial causes
Source: PLoS One. 2020 Apr 29;15(4):e0231314. doi: 10.1371/journal.pone.0231314 (PMC7190098; doi:10.1371/journal.pone.0231314)
Supplement: S1 Data — (DOCX) [file pone.0231314.s003.docx]

Online Supplemental Materials: Experimental Materials

Pilot Study 1A

Thank you for participating in this study. Please enter your 5-digit phone number below. This should match the number that you gave the experimenter earlier. 


We will use this number as the participant ID. At the end of the data collection process, we will randomly choose one of the numbers and email the entire pool that winning number. This procedure ensures that your responses will remain anonymous throughout the data collection process.

________________________________________________________________

In today's experiment, we are interested in cognitive reflex under time pressure. Please read the instructions carefully as you go through the questions.

There are three "Where's Waldo?" games that you can play. The rule is simple: your goal is to find Waldo (the character shown below) as fast as you can. You have up to 30 seconds per game, after which the survey page will self-advance. If you find Waldo before the time is up, click on ">>" to move to the next page.  


Click on ">>" when you are ready to begin:  

Game #1:

<Where’s Waldo Image here>

Did you spot Waldo?

- Yes, I found Waldo
- No, I did not find Waldo

How confident are you in your answer?

- None at all
- A little
- A moderate amount
- A lot
- A great deal

Game #2: 

 <Where’s Waldo Image here>

Did you spot Waldo?

- Yes, I found Waldo
- No, I did not find Waldo

How confident are you in your answer?

- None at all
- A little
- A moderate amount
- A lot
- A great deal

Game #3: Make your choices below: 

<Where’s Waldo Image here>

Did you spot Waldo?

- Yes, I found Waldo
- No, I did not find Waldo

How confident are you in your answer?

- None at all
- A little
- A moderate amount
- A lot
- A great deal

At this time, we would like to share an important message with you. Please read the following carefully. 
 
We are at a critical juncture.  The impact of most public and private sector policies depends critically on the behavior of individuals, groups, and organizations. A better understanding of policy implications and consequences firmly depends on the collaboration and cooperation of various stakeholders, such as social scientists, policymakers, practitioners, and **you**. 

 Your involvement in behavioral-based research help to promote the thoughtful application of rigorous, empirical behavioral science in ways that serve the public interest. By participating in experiments, you help to provide innovative and effective solutions that address current challenges facing policymakers today. 

 There are many ways you can get involved, beginning with signing a pledge. 

Please read the following petition regarding behavioral scientific research, and sign if you agree. Otherwise, leave this blank and move to the next page. 

 To create a better tomorrow, we must start today and draft evidence-based policies. Investing time, focus, and money in understanding the social and psychological implications of public and private policies is crucial in their eventual effectiveness. 
Join us at the Behavioral and Science Policy Association (behavioralpolicy.org) in helping to develop a rigorous, comprehensive, and evidence-based behavioral research. No matter what you do, let your actions be seen.

**PARTICIPANTS ARE THEN RANDOMLY ASSIGNED TO ONE OF THREE CONDITIONS: “LIKE,” “INITIALS,” or “SELF-OTHER INITIALS”**

**Like Condition**

Take the pledge by clicking on the Like Button below

- I pledge to support this endeavor
- I do not wish to sign this pledge

**Self-Initial Condition**

Take the pledge by typing your initials below
 
________________________________________________

**Self-Other Initials Condition**

Take the pledge by signing your name with the cursor in the space below.

**-----------------------------------------------------------------------------------------**

**DEPENDENT VARIABLE**

There are other ways you can help to advance behavioral research here at UVA


One challenge that behavioral scientists face is the paucity of willing participants. Participants are crucial in allowing researchers to understand and analyze decision-making models. The issue is that students at UVA are often unaware of the research opportunities around grounds. 


As a member of this community, you have insights into why your fellow students may not wish to participate, and how we can motivate more students to get involved. We would love to hear your thoughts. Please list each idea in the form below. For ease of interpretation, please enter each unique thought in a new line. Feel free to list as many as you like.

- Idea 1 ________________________________________________
- Idea 2 ________________________________________________
- Idea 3 ________________________________________________
- Idea 4 ________________________________________________
- Idea 5 ________________________________________________
- Idea 6 ________________________________________________
- Idea 7 ________________________________________________
- Idea 8 ________________________________________________
- Idea 9 ________________________________________________
- Idea 10 ________________________________________________

| Page Break |  |
| --- | --- |

| 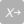 |
| --- |

It is helpful for us to know a little more about you. Please answer the following questions.

What is your gender?

________________________________________________________________

Age

________________________________________________________________

Thank you. Before you return to the experimenter desk,

**Pilot Study 1B**

We would like to ask for your opinion on a phenomenon that is quite common in our society today- Slacktivism
 
Slacktivism - a phenomenon in which people show support for a cause on social media without following up with actual behaviors that contribute to the cause - is becoming more and more prevalent in our society today. For instance, people would sign internet petitions (including liking pledges on Facebook) to feel that they have done something good without getting out of their chair.
 
The concern that slacktivism could encroach on meaningful support has led, for instance, to UNICEF Sweden’s “Likes Don’t Save Lives” campaign

Do you personally know people who have committed slacktivism in the past six months?

- Definitely not
- Might or might not
- Definitely yes

In general, how prevalent do you think slacktivism is?

- Never


- Very prevalent

How serious of an issue do you think slacktivism is in our society today?

- Not at all


- A great deal

Below are some reasons that may have contributed to people not following through with their commitment after making an online pledge. Some may have more explanatory power than others.


Now, for each of the reasons listed above, we would like you to indicate how much it contributes to slacktivism

|  | Not at all |  |  |  | Very much so |
| --- | --- | --- | --- | --- | --- |
| People feel less guilty for breaking online pledges and petitions |  |  |  |  |  |
| People feel less accountable to the pledges and petitions they sign online |  |  |  |  |  |
| There are too many online pledges and petitions around |  |  |  |  |  |
| Online pledges and petitions are less emotionally appealing |  |  |  |  |  |
| People often question the veracity of the online pledges and petitions |  |  |  |  |  |

Now,  rank order them based on the extent to which they explain why slacktivism occurs, with 1 being the most likely explanation and 7 being the least likely explanation.

______ People feel less guilty for breaking online pledges and petitions

______ People feel less accountable to the pledges and petitions they sign online

______ There are too many online pledges and petitions around

______ Online pledges and petitions are less emotionally appealing

______ People often question the veracity of the online pledges and petitions

Please indicate the gender you identify with.

- Male
- Female
- Other ________________________________________________

Your age:

________________________________________________________________

Do you have any comments for the researchers?

**Study 2**

During today's study, you will be reading some content and answering questions about it. It is important that you read each sentence carefully, as some of the questions later on will be asking you about what you read. If you are not able to do this, please close out of the survey now.

Please click the >> button to begin.

We would like to tell you about a issue affecting more than 12 million kids in the US today: Child Hunger

  **The Issue of Child Hunger in America:**
   According to the latest findings, **1 in 5 children in the U.S. lack proper nutrition and access to food at some point during the year**. The number escalates during summer. When school closes, about 18 million children lose access to free meals that are provided by the schools.

 For the more than **16 million kids** in the U.S. facing hunger, getting the energy they need to learn and grow can be a daily challenge. Hunger is associated with stomachaches, headaches, and a decreased ability to focus. These physical and cognitive consequences can cause hunger children to begin life at a serious disadvantage.  And as they grow up, kids struggling to get enough to eat are more likely to have problems in other social situations. Kids who don’t get enough to eat are also more likely to miss school and receive poor grades.  

Several nonprofits in the US are working to help eliminate child hunger in the U.S. Below are what they suggest you can do:

There are many ways you can get help. You can volunteer at a local food bank, organize a fundraising event, or donate money and other tangible resources. You can also help by becoming a vocal advocate for children who do not have enough to eat. Raise awareness of this issue in your community. Any step you take to help raise awareness of children in need can change a child's life for the better. 

Any action you take will work toward the same goal - to confront child hunger and give our future generations the nourishment they need to thrive. Let us join efforts and commit to help raise awareness for the cause in the next few weeks.

**PARTICIPANTS ARE THEN RANDOMLY ASSIGNED TO ONE OF THREE CONDITIONS: “SELF-INITIALS,” “SELF & TOP-OF-MIND INITIALS” or “SELF-DEDICATION INITIALS” THEY COULD HAVE ALSO CLICKED ON “I DO NOT WISH TO TAKE THE PLEDGE” TO INDICATE THAT THEY WISH TO OPT OUT OF THE PLEDGE.**

**SELF-INITIALS Condition**

    Please sign the pledge by entering your initials

__ I do not wish to take the pledge
   *​*

**SELF & TOP-OF-MIND INITIALS**

Think of someone who comes to mind, then sign the pledge with both your initials plus the initials of that person.

__ I do not wish to take the pledge

**SELF-DEDICATION INITIALS**

Think of a person who is extremely important to you and helped you become the person you are today, then dedicate the pledge that person with both your own initials plus the initials of that person.

__ I do not wish to take the pledge

**DEPENDENT VARIABLE**

Any action you can take would help with ending child hunger in the U.S. Please take some time and list the concrete steps that you will take. You can list as many or as few steps as you wish below.

Please indicate the gender you identify with below.

- Male
- Female
- Other ________________________________________________

Your age

________________________________________________________________

**Study 3**

During today's study, you will be reading some content and answering questions about it. It is important that you read each sentence carefully, as some of the questions later on will be asking you about what you read. If you are not able to do this, please close out of the survey now.

We would like to tell you about a nonprofit, U.S. based foundation: Boys & Girls Clubs of America

**About Boys & Girls Clubs of America
 ​**
 BGCA aims to enable all young people, especially those who need us most, to reach their full potential as productive, caring, responsible citizens. By designing innovative programs, local chapters of BGCA empower youth to excel in school, become good citizens and lead healthy, productive lives. Each year, local chapters of Boys & Girls Clubs help millions of kids and teens develop essential skills, make lasting connections and have fun. Club members experience: A safe place to learn, play and grow Supportive relationships with caring mentors Enriching programs, experiences and activities 
According to a 2017 study, **73%** of low-income members ages 12 to 17 who attend the Club regularly say they earn mostly As and Bs, compared with 69% of low-income youth nationally. **91%** of Club ninth graders report abstaining from alcohol, compared to 77% of ninth graders nationally.​

Founded in 1906, BGCA recently celebrated its Centennial year of providing hope and opportunity to young people across the country.
 
There are many ways you can get involved in BGCA. You can volunteer at a local chapter, organize a fundraising event, or donate money and other tangible resources. Any step you take to help raise awareness of children in need can change a child's life for the better. 


Any action you take will work toward the same goal - to strengthen and empower children in need. Join Boys and Girls Clubs of America campaign by committing yourself to help raise awareness for the foundation in the next few weeks.

**PARTICIPANTS ARE THEN RANDOMLY ASSIGNED TO ONE OF THREE CONDITIONS: “SELF-INITIALS,” “SELF & RANDOM INITIALS** **” or “SELF-DEDICATION INITIALS”**

**SELF-INITIALS Condition**

    Please sign the pledge by entering your initials

__ I do not wish to take the pledge
   *​*

**SELF & RANDOM INITIALS**

  Please sign the pledge by entering your initials + the letters shown below,

__ I do not wish to take the pledge
   ​

**SELF-DEDICATION INITIALS**

Think of a person who is extremely important to you and helped you become the person you are today, then dedicate the pledge that person with both your own initials plus the initials of that person.

__ I do not wish to take the pledge

**DEPENDENT VARIABLE**

Please read the following statements and indicate how much you agree or disagree.

|  | Strongly disagree |  | Neither agree nor disagree |  | Strongly agree |
| --- | --- | --- | --- | --- | --- |
|  |  |  |  |  |  |
| I will tell other people about the Boys and Girls Club and what they do |  |  |  |  |  |
| I feel very committed to missions of the Boys and Girls Club  I feel very connected to the children being served by the Boys and Girls Club |  |  |  |  |  |

Please indicate the gender you identify with below.

- Male
- Female
- Other ________________________________________________

Your age

________________________________________________________________

I
